# The European NUTS-level election dataset: A tool to map European electoral geography

**DOI:** 10.1177/13540688221083553

**Published:** 2022-04-15

**Authors:** Dominik Schraff, Ioannis Vergioglou, Buket Buse Demirci

**Affiliations:** 111977 Center for Comparative and International Studies, ETH, Zurich, Switzerland

**Keywords:** Election data, Europe, NUTS, dataset, subnational, regions

## Abstract

Datasets on subnational election results in Europe frequently do not match with regional statistics available for cross-national research, mainly because territorial statistical units change over time and do not map onto the national electoral districts. This hinders consistent comparative research across time. This research note introduces EU-NED, a new dataset on subnational election data that covers national and European parliamentary elections for European countries over the past 30 years. EU-NED’s major contribution is that it provides election results on disaggregated levels of the statistical territorial units used by Eurostat with an unprecedented consistency and temporospatial scope. Moreover, EU-NED is integrated with the Party Facts platform, allowing for a seamless integration of party-level data. Using EU-NED, we present first descriptive evidence on the European electoral geography and suggest avenues of how EU-NED can facilitate future comparative political science research in Europe.

## Introduction

Subnational variation in electoral outcomes is of central interest to comparative politics and the geographic polarization of electoral maps has recently received increasing attention (Dijkstra et al., 2019; Rodden, 2010; Schraff, 2019; Stockemer, 2017; Winkler, 2019). Disaggregated election data is used to improve our understanding of manifold political outcomes and processes. For instance, subnational electoral outcomes are central for ongoing research on radical right party success (Patana, 2020). Regional electoral data is also useful to investigating the effects of societal changes on political polarization (Winkler, 2019). Moreover, comparative data on subnational election outcomes helps to advance the long-standing research agenda on the nationalization and Europeanization of party systems (Caramani, 2011; Schakel, 2013) and the diffusion of electoral trends across borders (Börzel and Risse, 2012). Finally, comparative data on regional voting results are highly relevant for the comparative literature on a geography in anti-globalization preferences and Euroscepticism (Colantone and Stanig, 2018).

Despite the omnipresence of subnational context in European electoral studies, current comparative data is very limited in its geographic and temporal coverage. In fact, currently no readily available dataset consistently provides election results on the level of European territorial statistical units. This is a great obstacle for comparative research, as most socio-economic statistics are provided on the level of territorial statistical units. Eurostat, for instance, provides a wealth of statistical data (cf. Eurostat Regional Database) on the so-called NUTS (nomenclature of territorial units for statistics) regions. These territorial units are widely used for subnational comparative social science research, including the fields of regional studies (e.g., Dijkstra et al., 2019) economics (e.g., Becker et al., 2010; Crespo-Cuaresma et al., 2014), political science (e.g., Colantone and Stanig, 2018; Dellmuth and Chalmers, 2018), and sociology (e.g., Heidenreich and Wunder, 2008). The statistical territorial units frequently have social effects, making them a relevant unit of analysis. For instance, Debus et al. (2011) provide an intriguing study on how the NUTS administrative units shape political outcomes by demonstrating that regions from the same NUTS area form similar government coalitions.

Despite the relevance of NUTS statistical units, electoral data for this unit of analysis is surprisingly patchy and unconsolidated. Often, data is not available on the NUTS level or can only be accessed at very high levels of aggregation. Eurostat’s NUTS 0 and NUTS 1 codes usually refer to the national level or a few grand regions. The NUTS 2 and NUTS 3 codes, in contrast, capture more disaggregated subnational statistical units. For instance, Germany has the largest number of NUTS 3 regions with 401 districts (*Kreise or Kreisfreie Städte*) but only 16 regions at the NUTS 1 level (the federal states, *Länder*). Meanwhile, most other EU states report the national level as NUTS 1. In its regional database, Eurostat currently reports statistics for 281 regions at NUTS 2 and 1348 regions at NUTS 3 level.^1^

Despite the availability of socio-economic and demographic data according to the NUTS classification, some of the most prominent databases for election results, such as the European Election Database (EED) or the Constituency-Level Elections Archive (CLEA), do not systematically provide election outcomes on territorial statistical units (Klingemann et al., 2006; Kollman et al., 2019a). EED sometimes does not provide NUTS-level results due to difficulties with the recoding of official election data, frequently opting for data on electoral constituencies (e.g., Ireland) or for the more highly aggregated NUTS 1/2 level units (e.g., Belgium). Moreover, EED has not been updated in almost a decade.

CLEA provides election results on the level of electoral constituencies and the Georeferenced Electoral Districts (GRED) project enables the spatial visualization and analysis of CLEA data (Kollman et al., 2019b) However, electoral constituencies are frequently not aligned with territorial statistical units, and neither CLEA nor GRED account for most electoral constituency boundary changes over the years. For instance, only the 2010 electoral constituency borders of the UK are currently included in GRED, ignoring boundary changes across time. This renders many localities in the UK unfit for subnational political comparison using CLEA/GRED data across years since the differences in the pre-2010 and the post-2010 electoral constituency boundaries cannot be accounted for. Recoding disaggregated constituency data can be very tedious as constituencies and territorial units often change over time. Moreover, in some countries electoral constituencies are larger than NUTS 2 or NUTS 3 regions, which makes the constituency-level data reported in the CLEA database inadequate for studies of territorial statistical units.

The consolidated election data on NUTS statistical regions we present in this research note takes full advantage of the subnational statics provided by Eurostat. Other projects, like the Quality of Government data, use a similar strategy by collecting corruption data on the NUTS level and combining it with Eurostat’s regional data (Charron et al., 2020). The NUTS scheme, however, also has some disadvantages that researchers should consider. We acknowledge that the political and administrative relevance of NUTS regions differs across member states. While the NUTS classifications are purely statistical and have no administrative function in Latvia, in other countries such as Germany and Italy, the NUTS 3 regions do correspond to important administrative units. Projects like the regional authority index suggest that the politically important regions are often not on the NUTS 3 level, but the larger NUTS2 or NUTS1 level (Hooghe et al., 2010). However, we think that it is up to the researchers to decide which level of aggregation is most suitable for a given research question. We have collected data on the most disaggregated NUTS level available. Researchers then can always decide to aggregate the data as they see fit.

Another shortcoming of the NUTS regions is that even the most disaggregated level (NUTS 3) can lack detail in urban areas. The NUTS classification provides a rather detailed picture for less populated areas, while it lacks detail in more densely populated areas.^2^ However, the overrepresentation of less populous areas is not unique to NUTS-level electoral data. Even when one gathers and analyzes data at the electoral constituency level, some areas can have far more electors than others. To ameliorate this problem, one could, for instance, collect and analyze election data at the polling station level. However, there will be no comparative socio-economic data at such a disaggregated level across Europe, since no centralized source as Eurostat exists at that polling station level. While the current NUTS classification is not ideal, it is the best we have at our disposal now for comparative research on the subnational level.

To ease comparative research on subnational electoral results, this research note presents the European NUTS-Level Election Dataset (EU-NED). EU-NED provides national and European parliamentary election results on the level of NUTS2 and NUTS3 administrative units for 30 European countries over the period 1990-2020. EU-NED covers electoral data for over 950 parties nested in about 1200 European regions. For all countries and elections, regional units are harmonized on the 2016/2021 NUTS classification scheme.^3^ We provide votes for all parties reaching over one percent of the national vote share or exceeding one percent in a specific region. Smaller parties are put into an “other” category. If available, EU-NED includes Party Facts codes (Döring and Regel, 2019), which allows for seamless merging of party-level data. With this, EU-NED greatly facilitates comparative research on subnational context and election outcomes in Europe.

This research note is structured as follows. First, we shortly summarize the data collection process underlying the EU-NED database. A more detailed picture can be gained via the accompanying dataset codebook. Second, we present first descriptives to map the subnational electoral geography under European integration over the past three decades, putting a focus on patterns of far-right, Eurosceptic, and populist vote shares. Finally, this research note provides some suggestions for future avenues of research enabled by EU-NED.

## Harmonizing subnational election data across Europe

The major challenge for compiling subnational election data across Europe emerges from inconsistencies between electoral constituencies and territorial statistical units. These inconsistencies can emerge from a geographical mismatch, temporal changes in the territorial units, or temporal changes in electoral districts. In the following, we shortly summarize how EU-NED addresses these challenges.

Especially in proportional electoral systems with large constituencies, for example, the Netherlands, one needs to collect geographically disaggregated election results on a level that resembles the NUTS classification. In many cases, we avoid mismatches between geographical units used in election data and the NUTS scheme by collecting time-series data on highly disaggregated election results from the national authorities. This data is frequently on the level of local administrative units (LAU), such as municipalities. By relying on time-series data on a level of aggregation below the NUTS 2 or 3 units, we can directly aggregate to today’s NUTS classification.

Still, a major coding effort emerges from country-specific changes in LAU-level administrative units. In 2007, for instance, Denmark reduced its numbers of municipalities from 271 to 98, which also led to a reform of the Danish NUTS classification.^4^ It therefore is difficult to find data on the current 11 Danish NUTS 3 regions for any election prior to 2007, as the pre-2007 LAU units do not directly map onto today’s NUTS scheme. For EU-NED, we collected pre-2007 election results for the 271 historic municipalities. Using a map of the old municipalities and today’s NUTS 3 regions, we spatially matched the old municipalities to today’s NUTS 3 regions and aggregated the election results accordingly. This worked well as the old municipalities nest within today’s NUTS 3 boundaries.

To ensure valid electoral data, we refrained from aggregating local-level data if subnational units did not nest well within today’s NUTS structure. If the most disaggregated territorial units in the electoral data crosscut several NUTS boundaries, valid recoding into NUTS-level results was not feasible.^5^ For some countries, therefore, NUTS 3 aggregation was not viable and the electoral data we provide here is on the NUTS 2 level. For instance, the most disaggregate level for which the United Kingdom provides election results is the level of electoral constituencies, which frequently crosscut boundaries of NUTS 3 regions.^6^ UK constituencies, however, nest quite well into the larger NUTS 2 areas. In such instances, EU-NED only provides data on the NUTS 2 level. Table 1 lists all countries included in the EU-NED database and the corresponding NUTS level the electoral data could be compiled on.

**Table 1.** Level of disaggregation in the EU-NED electoral data.

Country	Elections covered	Smallest NUTS level available	Number of NUTS units
Austria	1990, 1994, 1995, 2002, 2006, 2008, 2013, 2017, 2019	3	35
Belgium	1991, 1995, 1999, 2003, 2007, 2010, 2014, 2019	2	11
Bulgaria	2005, 2009, 2013, 2014, 2017	3	29
Croatia	2011, 2015, 2016, 2020	3	22
Cyprus	1991, 1996, 2001, 2006, 2011, 2016	3 (same as NUTS 1/2)	1
Czechia	2002, 2006, 2010, 2013, 2017	3	14
Denmark	1990, 1994, 1998, 2001, 2005, 2007, 2011, 2015, 2019	3	11
Estonia	2003, 2007, 2011, 2015, 2019	3	5
Finland	1983, 1987, 1991, 1995, 1999, 2003, 2007, 2011, 2015, 2019	3	19
France	1993, 1997, 2002, 2007, 2012, 2017	3	101^a^
Germany	1990, 1994, 1998, 2002, 2005, 2009, 2013, 2017	3	401
Greece	1990, 1993, 1996, 2000, 2004, 2007, 2009, 2012 (June), 2015 (September), 2019	3	46^b^
Hungary	1990, 1994, 1998, 2002, 2006, 2010, 2014, 2018	3	20
Ireland	1992, 1997, 2002, 2007, 2011, 2016, 2020	2	3
Italy	1992, 1994, 1996, 2001, 2006, 2008, 2013, 2018	3	110
Latvia	2002, 2006, 2010, 2011, 2014, 2018	3	7
Lithuania	2000, 2004, 2008, 2012, 2016, 2020	3	11
Luxembourg	1994, 1999, 2004, 2009, 2013, 2018	3	1
Malta	2003, 2008, 2013, 2017	3	2
Netherlands	1994, 1998, 2002, 2003, 2006, 2010, 2012, 2017	2	12
Norway	1993, 1997, 2001, 2005, 2009, 2013, 2017	3	19
Poland	2001, 2005, 2007, 2011, 2015, 2019	2	18
Portugal	1991, 1995, 1999, 2002, 2005, 2009, 2011, 2015, 2019	3	25
Romania	2004, 2008, 2012, 2016, 2020	3	42
Slovakia	2006, 2010, 2012, 2016, 2020	3	8
Slovenia	2004, 2008, 2011, 2014, 2018	1	1
Spain	1993, 1996, 2000, 2004, 2008, 2011, 2015, 2016, 2019	3	52^c^
Sweden	1991, 1994, 1998, 2002, 2006, 2010, 2014, 2018	3	21
Switzerland	1991, 1995, 1999, 2003, 2007, 2011, 2015, 2019	3	26^d^
Turkey	2002, 2007, 2011, 2015, 2018	3	81
United Kingdom	1992, 1997, 2001, 2005, 2010, 2015, 2017, 2019	2	41

^a^Except for the elections of 1993 and 1997, for which there are only 96 regions. These elections do not report information on the overseas departments of France.

^b^The regions of Athens and Thessaloniki are reported at the NUTS 2 level.

^c^The islands of Balears and Canary Islands are reported at the NUTS 2 level.

^d^In 1999, the Canton of Obwalden held no parliamentary election because there was only one candidate. They were automatically given a seat. The same situation happened in Nidwalden in the 2007 election.

We further harmonized our data on the party level by providing the native and English party labels. A complete list of parties is provided in the codebook. If available, EU-NED includes the party identifiers used on the Party Facts platform (Bederke et al., 2020). These identifiers are sometimes missing, as Party Facts does not assign codes when parties are electorally irrelevant. This makes sense since available party-level data, such as expert ratings, usually focuses on the relevant parties (e.g., with at least 1-3 percent vote shares).

The data structure of EU-NED is based on a region-party-election year unit of analysis. We have opted for election years, as EU-NED’s main goal is to maximize the match with regional statistical data.^7^Table 2 provides an impression of the data structure. The EU-NED website (www.eu-ned.com) will provide a number of dataset versions to cater different researchers’ interests, as well R code to reshape the data and merge Eurostat’s regional statistics. EU-NED is also archived on the Harvard Dataverse, and we will update this repository as new dataset versions are created (https://doi.org/10.7910/DVN/IQRYP5). The standard EU-NED dataset version follows the data structure of Table 2 and provides the regional results for each region-party-election year. The EU-NED panel dataset version expands the standard dataset to a yearly panel ranging from 1990-2020, extrapolating election results from one election year to the next. This dataset version is used to present the descriptives on temporal trends in the next section. Finally, EU-NED comes as a regional structural dataset, merging all regional Eurostat statistics to the region-party-election year electoral data. These different dataset versions can be seen as a service to the community to facilitate comparative research on the European electoral geography.

**Table 2.** Snapshot of EU-NED database structure.

nuts2016	regionname	type	year	party_abbreviation	party_english	party_native	partyfacts_id	partyvote	electorate	totalvote	validvote
AT111	Mittelburgenland	Parliament	1990	FPO	Freedom Party of Austria	Freiheitliche Partei Osterreichs	463	2095	30,187	26,871	26,205
AT111	Mittelburgenland	Parliament	1990	GRUNE	The Greens—The Green Alternative	Die Grunen—Die grune Alternative	1659	527	30,187	26,871	26,205
AT111	Mittelburgenland	Parliament	1990	OTHER	NA	NA	NA	51	30,187	26,871	26,205
AT111	Mittelburgenland	Parliament	1990	OVP	Austrian People’s Party	Osterreichische Volkspartei	1329	10,186	30,187	26,871	26,205
AT111	Mittelburgenland	Parliament	1990	SPO	Social Democratic Party of Austria	Sozialdemokratische Partei Osterreichs	1384	13,180	30,187	26,871	26,205
AT111	Mittelburgenland	Parliament	1990	VGO	United Greens Austria	Vereinte Grune Osterreichs	1170	166	30,187	26,871	26,205
AT111	Mittelburgenland	Parliament	1994	FPO	Freedom Party of Austria	Freiheitliche Partei Osterreichs	463	3591	30,819	26,923	26,345
AT111	Mittelburgenland	Parliament	1994	GRUNE	The Greens—The Green Alternative	Die Grunen—Die grune Alternative	1659	822	30,819	26,923	26,345
AT111	Mittelburgenland	Parliament	1994	LIF	Liberal Forum	Liberales Forum	605	606	30,819	26,923	26,345

## The electoral geography of Europe, 1990-2020

To demonstrate the potential of EU-NED, we present descriptives on the European electoral geography over the time of the EU enlargement process and European crisis events, such as the Euro- and migration crises. As EU-NED is integrated with Party Facts, one can directly merge party-level information to measure party positions or other types of party-level characteristics. For our purposes here, we merged EU-NED with the PopuList (Rooduijn et al., 2019), a dataset that has recently been integrated with the Party Facts platform. The PopuList is a prominent dataset that uses expert judgement to classify European parties as populist, far-right, and Eurosceptic. We can use EU-NED alongside the PopuList classification to present the electoral geography in populist, far-right, and Eurosceptic party support.

**Figure 1.**
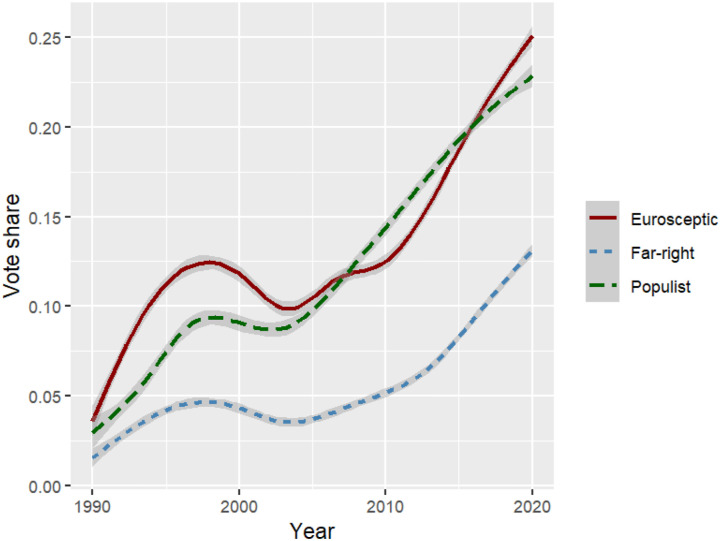
European trend in Eurosceptic, far-right, and populist electoral support. Note: The figure depicts the yearly vote share of Eurosceptic, far-right, and populist parties recorded in the EU-NED database. Party coding relies on the PopuList (Rooduijn et al., 2019).

In Figure 1, we present the aggregated temporal trend in populist, far-right, and Eurosceptic party support as measured with EU-NED. As probably many scholars of European politics would suspect, we see a substantial increase in the vote shares of far-right, Eurosceptic and populist parties. As EU-NED covers all EU member states and some closely associated ones (e.g., Norway and Switzerland), it can provide an externally valid presentation of how Eurosceptic, populist, and far-right voting have developed over the course of EU integration. Eurosceptic party support saw a strong increase in the 1990s, remained stable over the 2000s and strongly rose again as the Eurocrisis unfolded in 2008/9. The trend in populist party support closely follows the Euroscepticism trend. However, populist support remained below Eurosceptic vote shares in the 1990s, but converged with levels of Euroscepticism during the 2000s. Far-right party support remains rather low until 2010, but then strongly rises as the Eurocrisis and migration crisis hit Europe. This suggests that the rise of far-right voting in the European Union is a more recent trend than the rise of Eurosceptic or populist voting.

**Figure 2.**
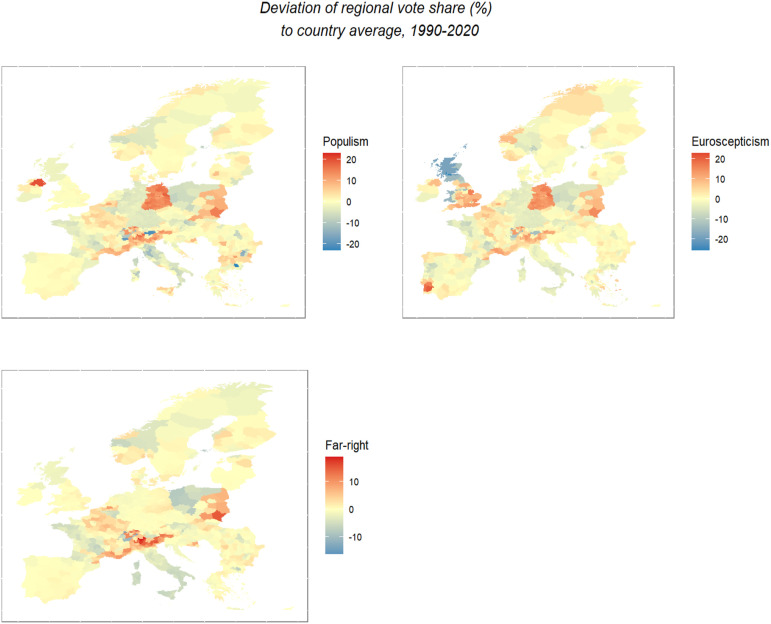
Within-country variation in the European electoral geography. Note: The maps present a region’s vote share relative to the respective country’s average vote share. This produces a visualization of the within-country variation in populist, Eurosceptic, and far-right voting. The deviations for the Eastern European member states rely on data from accession in 2004 onwards. Party coding relies on the PopuList (Rooduijn et al., 2019).

Figure 2 now turns to the electoral geography in populist, far-right, and Eurosceptic party support. It pools regional vote shares over the whole observation period of 1990–2020, therefore providing a picture of the average electoral geography from the past three decades.^8^Figure 2 presents regional vote shares as deviations from parties’ average national vote share. This brings the regional values on a similar scale and maximizes the visibility of subnational (within-country) variation. We see that subnational variation is sizeable in many countries such as France, Italy, or Poland. However, not all countries show strong geographic variation. Spain, for instance, has a limited geographical variation in the electoral geography over the past three decades and just started to polarize geographically in more recent years. In other countries, geographic variation depends on the types of parties. The United Kingdom and Portugal, for example, shows strong regional polarization with respect to Eurosceptic party support, but not regarding populism or far-right party support. Contrarily, some Eastern member states, such as Bulgaria or Romania, show more pronounced regional polarization with respect to populism and far-right support, but smaller spatial divides with respect to Eurosceptic party voting.

**Figure 3.**
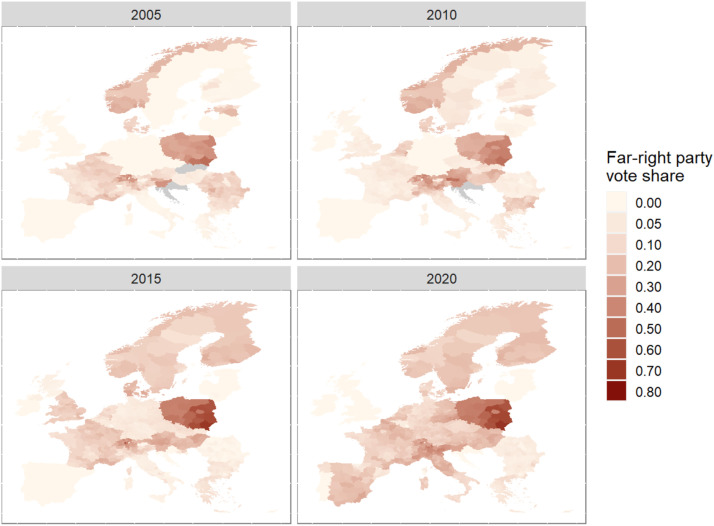
Temporal change in regional far-right vote shares. Note: Party coding relies on the PopuList (Rooduijn et al., 2019).

Figure 3 maps the temporal changes in far-right voting.^9^ Here, we focus on the trend since the EU’s Eastern enlargements and the periods of the Eurozone and migration crises. Over the past 15 years, far-right party support has spread rather uniformly across most European states, from North to South and East to West. Today, the far-right has a solid base in most European states, with only a few exceptions in Portugal or the Baltics. However, as Figure 3 shows, the within-country geographic variation of far-right party support is visible in many states and gives rise to an emerging research agenda on the political geography of far-right voting. EU-NED is the first dataset that allows researchers to investigate this pattern with such a broad spatiotemporal scope.

## Avenues for future research with EU-NED

Analyzing the electoral geography across Europe promises to improve our understanding of manifold political processes. EU-NED provides a comprehensive foundation for comparative research on the electoral geography in Europe. Below, we shortly outline three avenues for future research that will be facilitated with EU-NED. We do not claim that these are the only or most relevant avenues for future research. Rather, we provide an outlook for research agendas that also reverberate in the descriptives provided above.

### The political geography of far-right party success

As Figure 3 shows, far-right voting has increased markedly in recent years and shows substantial regional variation within European states. Far-right electoral polarization along the lines of regional divides has recently received increasing attention in comparative politics. Fitzgerald (2018), for example, argues that local context conditions, such as socio-demographic homogeneity, are crucial determinants for understanding the success of far-right parties. Current evidence points towards a pronounced geography of a cosmopolitan versus communitarian divide fueled by immigration and economic transformations (Huijsmans et al., 2021; Maxwell, 2020; Patana, 2020). Most of these findings, however, rest on single-country studies.

EU-NED will be able to improve our comparative understanding of the nature of and cross-national differences in the electoral geography of the far-right. Here, one avenue for research lies in a deeper understanding of the diversity we can observe within countries’ electoral geographies. For instance, why does the geography of far-right voting sometimes appear to follow an economic divide and sometimes not (Carreras et al., 2019)? Subnational data on socio-economic as well as demographic variables are provided at the NUTS levels by Eurostat, which makes subnational analysis for European nations possible. By merging this disaggregate data with party-level characteristics and electoral results, EU-NED can enable researchers to provide a comprehensive picture of the subnational divide in far-right voting.

### Inequality and the polarization of the electoral geography

Some of the most important transformations European societies experience today have regional, context-dependent implications. Rising inequality, for example, reshapes living conditions across European regions, which appears to be channeled into political preferences and behaviors (Colantone and Stanig, 2018; Lipps and Schraff, 2020; Winkler, 2019). European integration is often argued to be a major cause of rising inequality, as integration has driven welfare state retrenchment as well as distributive asymmetries (Beckfield, 2019; Busemeyer and Tober, 2015). Yet, most of existing research does not connect the process of integration to the geospatial polarization in electoral behavior. This is because EU-wide data on the electoral geography has so far been missing. EU-NED promises to facilitate comparative research on the relationship between social transformations, geography, and the popular response.

### Europeanization of party systems and diffusion

Finally, EU-NED holds the potential to advance research on classical topics of the Europeanization of party systems (Caramani, 2011; Schakel, 2013). Fine-grained geospatial data facilitates research on the diffusion of electoral trends across the national borders of European democracies (Börzel and Risse, 2012). Yet, analyzing the diffusion of electoral trends requires temporal data. A streamlined dataset covering the past 30 years, such as EU-NED, is therefore uniquely suited for diffusion studies. Moreover, diffusion studies would like to uncover the conditions under which geographical spillovers take place. It is therefore important to merge the regional electoral panel data with socio-demographic statistics. Here, EU-NED is helpful as it directly aligns the subnational electoral data with Eurostat’s regional statistics.

## Conclusion

This research note introduced a new dataset to study the electoral geography of Europe. The European NUTS-Level Election Dataset (EU-NED) provides fine-grained subnational electoral data across all EU countries over the period of 1990-2020. It offers consistent coverage of election results for national and European parliamentary elections for Eurostat’s smallest territorial units, enabling the combination of electoral and regional socio-demographic data. Moreover, EU-NED is integrated with the Party Facts platform, permitting a seamless integration of party-level characteristics. In this note, we introduced the dataset and its data collection method, outlining how EU-NED overcomes the restriction of existing datasets on European election results. We presented first descriptives of the European electoral geography and provided suggestions for future research with EU-NED data.

We believe this dataset will improve the quality of comparative research on European politics through making party-level and subnational electoral data easily accessible for research. EU-NED is planned to be maintained for future elections.

## Notes

1. On January 1 2021, Eurostat moved from the 2016 NUTS version to the 2021 NUTS. EU-NED will be made available with 2021 NUTS codes as well.

2. We thank an anonymous reviewer for highlighting this issue.

3. Eurostat regularly revises the NUTS classification scheme. EU-NED is provided with the two recent versions—the 2016 and the 2021 NUTS classification. Changes from the 2016 to the 2021 version are minor. Only the NUTS areas in Norway were reformed substantially.

4. https://english.im.dk/responsibilities-of-the-ministry/economics-of-municipalities-and-regions/structural-reform [accessed on 28 September 2021]

5. In these instances, we always checked back with national authorities on which level valid aggregation is feasible.

6. https://commonslibrary.parliament.uk/research-briefings/cbp-8647/[accessed on 28 September 2021]

7. If two elections happen within the same year (e.g., Spain 2019), we only provide data for the second election.

8. Note that the Eastern European states enter the dataset around their accession to the European Union. Temporal coverage for these states currently starts from the mid-2000s onwards.

9. Note that data for Croatia and Slovakia are currently only available from the years after the countries joined the European Union. We are currently working on expanding the data towards earlier elections.
